# The Role of a Plant-Only (Vegan) Diet in Gastroesophageal Reflux Disease: Online Survey of the Italian General Population

**DOI:** 10.3390/nu15224725

**Published:** 2023-11-08

**Authors:** Gianluca Rizzo, Luciana Baroni, Chiara Bonetto, Pierfrancesco Visaggi, Mattia Orazzini, Irene Solinas, Giada Guidi, Jessica Pugliese, Giulia Scaramuzza, Filippo Ovidi, Irene Buselli, Massimo Bellini, Edoardo V. Savarino, Nicola de Bortoli

**Affiliations:** 1 Independent Researcher, 98121 Messina, Italy; drgianlucarizzo@gmail.com; 2 Scientific Society for Vegetarian Nutrition, 30171 Venice, Italy; 3 Section of Psychiatry, Department of Neurosciences, Biomedicine and Movement Sciences, University of Verona, 37134 Verona, Italy; chiara.bonetto@univr.it; 4 Division of Gastroenterology, Department of Translational Research and New Technologies in Medicine and Surgery, University of Pisa, 56126 Pisa, Italy; pierfrancesco.visaggi@phd.unipi.it (P.V.); m.orazzini4@studenti.unipi.it (M.O.); i.solinas1@studenti.unipi.it (I.S.); 27627122@studenti.unipi.it (G.G.); j.pugliese1@studenti.unipi.it (J.P.); g.scaramuzza@studenti.unipi.it (G.S.); f.ovidi2@studenti.unipi.it (F.O.); i.buselli2@studenti.unipi.it (I.B.); massimo.bellini@unipi.it (M.B.); nicola.debortoli@unipi.it (N.d.B.); 5 Division of Gastroenterology, Department of Surgery, Oncology and Gastroenterology, University of Padua, 35124 Padua, Italy; edoardo.savarino@unipd.it; 6 NUTRAFOOD, Interdepartmental Center for Nutraceutical Research and Nutrition for Health, University of Pisa, 56124 Pisa, Italy

**Keywords:** gastroesophageal reflux disease, GERD, plant-based diet, plant-only diet, vegan diet, heartburn, regurgitation, non-cardiac chest pain, lifestyle habits, Quality of Life, QoL, SF-36

## Abstract

The relationship between food and the pathophysiological mechanisms of gastroesophageal reflux disease (GERD) is unclear. There are few data on the impact of dietary habits on GERD symptoms and on the incidence of GERD in subjects undergoing plant-based diets. In this study, we investigated the association between diet and GERD, using data collected through an online survey of the Italian general population. In total, 1077 subjects participated in the study. GERD was defined according to the Montreal Consensus. For all subjects age, gender, body mass index (BMI), marital status, education, occupation, alcohol consumption, and smoking habits were recorded. All participants also completed the SF-36 questionnaire on Quality of Life. A total of 402 subjects (37.3%) were vegans and 675 (62.7%) non-vegans. The prevalence of GERD in the total population was 9%. Subjects with GERD-related symptoms recorded a worse quality of life according to SF-36 analysis (*p* < 0.05 for all dimensions). In multivariate analysis, after adjusting for confounders, participants undergoing a vegan diet had a significantly lower risk of GERD (OR = 0.47, 95% CI 0.28–0.81, *p* = 0.006). These findings should be taken into account to inform the lifestyle management of GERD.

## 1. Introduction

Gastroesophageal reflux disease (GERD) occurs when the passage of gastric contents back into the esophagus causes either mucosal damage or symptoms [1]. When GERD is defined as heartburn and/or acid regurgitation occurring at least weekly, its prevalence is less than 5% in Asia, and ranges from 10% to 20% in Western countries [2,3,4]. There is evidence that the prevalence of GERD has increased over the past two decades [4,5,6].

The main pathological mechanism is the passage of gastric contents into the esophagus and the dysfunction of the esophageal anti-reflux barrier. The former is primarily brought about by delayed stomach emptying and the creation of gastric acid pockets. The latter is mostly brought on by the lower esophageal sphincter’s (LES) malfunction. Among other things, there is an increase in the frequency of transient lower esophageal sphincter relaxation (TLESR) and a reduction in esophageal clearing mechanisms [7,8].

However, the reason for the increase in GERD and its complications is not yet clear. It is likely that the general change in dietary habits plays an important role: diets in Western countries are now mainly characterized by the consumption of sugars, fats, and animal foods instead of plant foods [9]. Many studies have indicated a relationship between the increasing prevalence of obesity and GERD [10,11]. Accordingly, it has been shown that a diet planned to induce weight loss decreases symptoms and PPI consumption in overweight/obese GERD subjects [10]. Few studies have investigated the role of different dietary patterns in the development of reflux symptoms, often leading to conflicting results [12,13]. The American College of Gastroenterology recommends that subjects with GERD reduce their intake of total fat, chocolate, alcohol, citrus fruits, tomato products, coffee, tea, and large meals, and make lifestyle changes, including quitting smoking and loosing body weight. It has been suggested that there is a potential difference in dietary style among patients with erosive and non-erosive GERD [14]. More recently, a potential role of functional foods seems playing some role in GERD management [15]. However, due to the paucity of evidence, routine global elimination of foods that may trigger reflux is not recommended for the treatment of all subjects with GERD [16,17,18]. To date, there are few data on the role of different dietary patterns on GERD symptoms, which affect the quality of daily life, interfering with physical activity, social life, sleep, and productivity at work [19,20,21]. According to previous guidelines, a negative impact on quality of life is a criterion for the diagnosis of GERD in subjects with frequent heartburn [22,23].

In this study, we investigated the association between a plant-only (vegan) diet and GERD-related symptoms after adjusting for socio-demographic characteristics, life habits, and health-related quality of life by using data collected through an online survey.

## 2. Materials and Methods

### 2.1. Data Collection

The INVITA study (INVestigation on ITAlians’ habits and health) uses an online survey launched on 26 July 2022, with the aim of cross-sectionally collecting data on the lifestyle, health status, and diet of the Italian general population. Participants were voluntarily recruited online by advertising the access link of the study through social media and newsletters. The exclusion criteria were age < 18 years, pregnancy or breastfeeding, and plant-based dietary restrictions (macrobiotic, fruit-based, raw-food, hygienist diets). The survey ensured anonymity and informed consent was obtained from all the participants. The online questionnaire was hosted by the Scientific Society for Vegetarian Nutrition (an Italian non-profit organization) in a dedicated application on the domain www.studioinvita.it (accessed on 26 July 2022) and could be accessed from computers, tablets, and smartphones. The data collected were downloaded and managed by data management personnel who had no possibility to identify study participants. This study was approved by the Bioethics Committee of the University of Pisa, Italy (Prot. N. 0116339/2021, approval date 29 September 2021).

### 2.2. Assessments

The dietary pattern (‘vegan’ or ‘non-vegan’) classification was established by categorizing participants who consumed at least one food item among meat, fish, poultry, dairy, or eggs as ‘non-vegan,’ and those who did not consume any food among meat, fish, poultry, dairy, or eggs as ‘vegan.’ GERD was diagnosed according to the Montreal consensus [23] by evaluating the presence of chest pain, regurgitation, and heartburn. Subjects were diagnosed as either having (GERD+) or not having (GERD−). To be considered as GERD-related, symptoms were required to have occurred two or more times per week over the previous 30 days. An ad hoc question about medications was used to classify those subjects who were controlling GERD symptoms with antiacids, histamine-2 blockers and/or proton pump inhibitors as GERD+.

The health-related quality of life was assessed by the self-reported Medical Outcomes Study 36-item Short Form Survey (SF-36; Italian version) [24]. The scale comprises 36 items. Item 1 asks participants to judge their health condition in general as excellent, very good, good, fair, or poor. Item 2 asks to rate their health in general compared to one year ago (from 1 ‘Much better now than one year ago’ to 5 ‘Much worse now than one year ago’). Items 3–12 describe how their health status could limit a series of activities usually performed during a typical day (vigorous activities such as running, lifting heavy objects etc.; moderate activities such as moving a table, pushing a vacuum cleaner, etc.; lifting or carrying groceries; climbing several flights of stairs; climbing one flight of stairs; bending, kneeling, or stooping; walking more than a mile; walking several blocks; walking one block; bathing or dressing yourself). Items 13–16 list (with an option of ‘Yes’ or ‘No’) some problems with work or other daily activities as a result of physical health in the past 4 weeks (cut down the amount of time spent on work or other activities; accomplished less than a subject would like; limited in the kind of work or other activities; difficulty performing the work or other activities). Items 17–19 ask (with an option of ‘Yes’ or ‘No’) about problems with work or other regular activities as a result of emotional problems in the past 4 weeks (cut down the amount of time spent on work or other activities; accomplished less than a subject would like; did not work or do other activities as carefully as usual). Item 20 ‘During the past 4 weeks, to what extent has your physical health or emotional problems interfered with your normal social activities with family, friends, neighbors, or groups?’ was scored from 1 ‘Not at all’ to 5 ‘Extremely’. Item 21 explores how much bodily pain was experienced during the past 4 weeks (from 1 ‘None’ to 6 ‘Very severe’), while item 22 asks how much pain interfered with the normal work (from 1 ‘Not at all’ to 5 ‘Extremely). Items 23–31 assess how participants felt during the past 4 weeks (very nervous, down in the dumps, calm and peaceful, with a lot of energy, etc.) by scoring from 1 ‘All of the time’ to 6 ‘None of the time’. Item 32 ‘During the past 4 weeks, how much of the time has your physical health or emotional problems interfered with your social activities?’ was scored from 1 ‘All of the time’ to 5 ‘None of the time’. Finally, items 33–36 ask participants to judge as true or false statements about their health (to become sick a little easier than other people; healthy as anybody I know; to expect health becoming worse; to have excellent health). All items were recorded so that a high score defined a more favorable health status. In addition, each item was scored on a range from 0 to 100 to represent the percentage of total possible score achieved. After that, items were averaged together to create 8 dimensions: general health (5 items), physical functioning (10 items), role limitations due to emotional problems (3 items), bodily pain (2 items), emotional well-being (5 items), role limitations due to physical health (4 items), energy/fatigue (4 items), and social functioning (2 items).

Moreover, ad hoc forms were used to collect sociodemographic characteristics and lifestyle habits: gender, age, marital status, education level, occupation, self-reported height and weight (BMI was computed by dividing weight in kilograms by height in meters squared), smoking history (yes/no), and alcohol consumption per month (1 alcohol unit, AU = 12 gr of pure alcohol, which corresponds to an average 330 cc of beer or 125 cc of wine or 80 cc of vermouth or 40 cc of liquor. ‘At risk’ consumption was defined as >60 AUs for males and >30 AUs for females [25,26]).

### 2.3. Statistical Analyses

Categorical variables were described as absolute numbers and percentages; continuous variables were summarized as means and standard deviations (SDs). Comparisons between groups were performed by Fisher’s exact test (4 cells) or Chi-square test (more than 4 cells) in the case of categorical variables, and by *t* test in the case of continuous variables. Subsequently, univariate logistic regression models with GERD+ as the dependent variable and each characteristic (dietary pattern and a set of possible confounding factors such as gender, age, marital status, education, occupation, BMI, alcohol consumption, smoking, and the 8 quality of life dimensions) as the independent variable were estimated to calculate unadjusted ORs. The characteristics that were found to be associated (at *p* < 0.05) with GERD+ entered the multivariate logistic regression model, returning adjusted ORs. All tests were two-tailed, with a significance level of 0.05. Analyses were performed by Stata 17 for Windows.

## 3. Results

**Figure 1:**
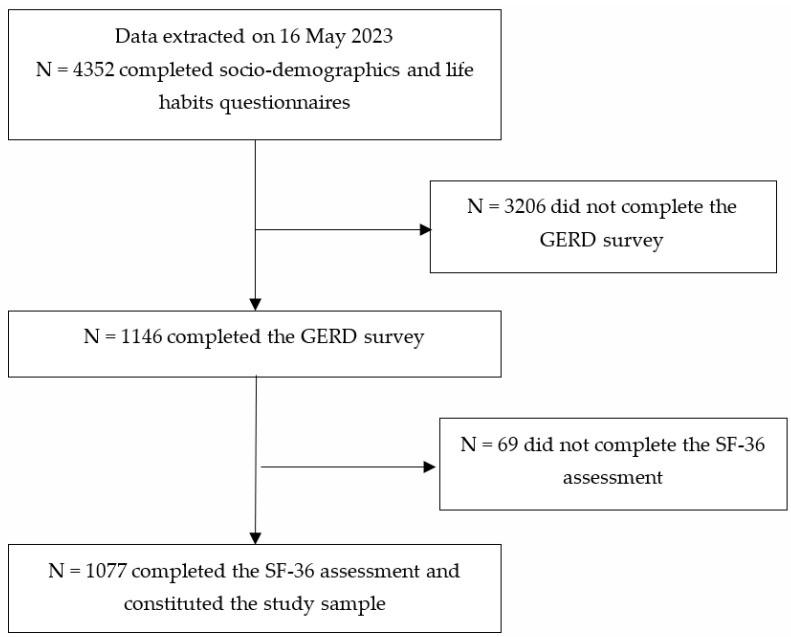
Flowchart of participants throughout the study.

At the time data were extracted (16 May 2023), 4352 subjects completed socio-demographics and life habits questionnaires. Of these, 1077 (24.7%) completed both the GERD survey and the SF-36 assessment and were included in the study (Figure 1).

A percentage of about 9% were found to have GERD symptoms and were categorized as GERD+. The number of participants in the sample giving information about medications were 929. In this sub-sample, the number of subjects taking antiacids, histamine 2 blockers, and/or proton pump inhibitors (PPI) were 16. Furthermore, 93% of participants were female, the mean age of the overall population was 37 ± 12 years, more than 60% were married, about 65% had a high education level (a degree or a post-degree), and more than 70% were employed. The mean BMI was 22.2 (SD 3.8) (Table 1, part a). By considering life habits (Table 1, part b), 4.9% declared a monthly alcohol consumption at risk, 9% were smokers, and 37.3% were vegans. By comparing socio-demographic characteristics and life habits between the study sample (n = 1077) and the subjects who did not complete the GERD survey or the SF-36 (n = 3275), age (37.1, SD 12.0 vs. 35.2, SD 11.8; *p* < 0.001 *t* test), vegan dietary pattern (37.3% vs. 31.7%; *p* < 0.001 Fisher’s test), and monthly alcohol consumption (no consumption 21.4% vs. 1.1%, low/moderate 73.7% vs. 90.8%, at risk 4.9% vs. 8.2%; *p* < 0.001 Chi-square test) were the only variables reaching a statistical significance. By considering the health-related quality of life (Table 1, part c), the mean scores for the eight dimensions ranged from 53.8 (SD 18.4) for Energy/fatigue to 94.6 (SD 9.9) for Physical functioning.

GERD+ subjects had a higher BMI (24.1, SD 5.4 vs. 22.0, SD 3.5; *p* < 0.001 *t* test), a lower education level (degree/post-degree 56.8% vs. 67.3%; *p* = 0.027 Fisher’s test), a lower percentage of vegan dietary pattern (24.2% vs. 38.6%; *p* = 0.005 Fisher’s test), and a higher percentage of smoking habit (16.0% vs. 8.4%; *p* = 0.022 Fisher’s test). All the health-related quality of life dimensions showed that the GERD+ group had mean scores lower than the GERD− group.

The unadjusted ORs estimated by univariate logistic regression models confirmed the association between GERD+ and BMI, education, dietary pattern, current smoking, and all the health-related quality of life dimensions (*p* < 0.05 for all) (Table 2).

These characteristics entered the multivariate logistic regression model ultimately providing adjusted ORs (adj-ORs) (Table 3). A higher BMI (adj-OR = 1.07, *p* = 0.007), smoking (adj-OR = 1.97, *p* = 0.039), a worse General health (adj-OR = 0.97, *p* = 0.001), and a worse Bodily pain (adj-OR = 0.98, *p* = 0.005) were significantly associated with GERD+ condition, while a vegan dietary pattern was inversely associated with GERD+ status (adj-OR = 0.47, *p* = 0.006).

## 4. Discussion

GERD is a very common disease, affecting about 1 billion people worldwide with some degree of variability according to the geographical location. In Europe, the prevalence of GERD is about 14.12% [3,27]. Typically reported risk factors are represented by sex, age, BMI, use of non-steroidal anti-inflammatory drugs, and smoking [3,27,28]. Additionally, diet is a potential risk factor for GERD symptoms; however, there is currently limited research on the impact of dietary choices on reflux symptoms [16,29]. The clinical diagnosis of GERD is based on the frequency of troublesome symptoms such as heartburn, regurgitation, and chest pain [23,30]. The recently updated version of the Lyon Consensus 2.0 suggests that only patients with typical symptoms (without clinical red signs) should be approached with a short empiric trial of proton pump inhibitors (PPIs) because the likelihood of GERD is quite high compared to atypical or extraesophageal presentations [31].

Our results showed a very strong association between some dietary choices and GERD: a plant-only (vegan) diet was inversely associated with the GERD+ condition (about halving the risk, compared to any other animal-based dietary patterns (OR = 0.47, 95% CI 0.28–0.81, *p* = 0.006)).

Moreover, we confirmed other established GERD risk factors, including smoking cigarettes (OR 1.97) and increased BMI (OR 1.07). In addition, the Quality-of-Life (SF-36) perception resulted lower in GERD+ subjects.

The American College of Gastroenterology guidelines [17] suggest, in the statement regarding lifestyle modifications for GERD treatment, avoiding trigger foods (indicated individually), reducing body weight for overweight and obese subjects, avoiding tobacco smoking, and head of bed elevation for subjects with nighttime symptoms. Despite the low level of evidence, the American College of Gastroenterology suggests cessation of foods that potentially aggravate reflux symptoms such as coffee, chocolate, carbonated beverages, spicy foods, and acidic foods such as citrus and tomatoes [11,17].

Only a few studies have evaluated the role of food components in the genesis of reflux symptoms, with conflicting results [9,12,18]. Moreover, eating animal food has been associated with a worsening of GERD symptoms. Similarly, a high-fat diet, including mainly animal fats, is considered a risk factor for the development of GERD complications such as Barrett esophagus [9,32,33].

Zalvan et al. suggested that a plant-based Mediterranean diet should be considered in the treatment of laryngopharyngeal reflux. A Mediterranean diet includes plant foods such as vegetables, bread and other grains, potatoes, beans, nuts and seeds, fresh fruit as the typical daily dessert, olive oil as the principal source of fat, dairy products (principally cheese and yoghurt), and fish and poultry consumed in low to moderate amounts, zero to four eggs consumed weekly, red meat consumed in low amounts, and wine consumed in low to moderate amounts, normally at mealtime [34].

Another study by Jung J.G. et al. suggested that a vegetarian diet may offer a protective effect for reflux esophagitis [35]. Similarly, Martinucci I. et al. [9] have shown that plant foods are associated with a lower number of reflux episodes, particularly acid refluxes, and with a reduced number of symptoms during the first postprandial hour. Unfortunately, these studies included a relatively small sample of individuals, and their findings warrant further investigation. Vegetarians may experience fewer symptoms of gastroesophageal reflux due to a typically healthier lifestyle [36], and some research has indicated that a vegetarian diet may be associated with improved mood and reduced stress [37]; these factors could potentially reduce reflux symptoms [38]. Nevertheless, it is yet to be determined whether subjects with GERD symptoms and related issues can benefit from adopting a vegetarian diet. In support of the potential anti-reflux effect of fiber, it was shown that fiber food improved heartburn symptoms in a randomized controlled trial [39]. The vegetarian diet is also rich in antioxidants and maintains a higher antioxidant vitamin status (vitamin C, vitamin E, ß-carotene) [40]. a chronic oxidative stress has been shown to contribute to the development of GERD [41,42], and diets high in vitamin C content were associated with a lower risk of GERD [43].

The determinant role of vegetables and fibers in the diet has been underlined in many different studies. A very elegant study provided from Houston team (US) discovered that a daily intake of more than 1.58 cups of vegetables and 0.18 cups of dark green vegetables per 1000 calories was associated with a lower risk of intestinal metaplasia in the esophagus (Barrett Esophagus, BE) [44].

Kubo et al., in a population-based case–control study conducted in the United States, observed that the consumption of veggies was associated with a lower risk of BE [45]. Similarly, a nice research study, conducted in Washington State with 170 hospitalized cases and 182 controls from the general population, showed that a global vegetable intake was linked to a 60–70% risk decrease for BE [46].

Anderson and colleagues found an inverse correlation between fruit and vegetable intake and the risk of complicated GERD [47]. However, consumption of leafy or dark green vegetables has consistently been linked to a lower risk of cancer [48,49,50]. Different reports have shown that dietary fibers are known to play a determinant role in the prevention of different gastrointestinal diseases such as constipation, hemorrhoids, colon cancer, gastroesophageal reflux disease, duodenal ulcer, and diverticulitis, as well in serious and systemic diseases such as obesity, diabetes, stroke, hypertension, and cardiovascular diseases [51,52,53].

A reduction in Quality-of-Life in GERD subjects has been reported in previous studies [54,55,56,57]. The QoL in patients with GERD-related symptoms was lower than that associated with untreated duodenal ulcer, angina, mild heart failure, diabetes, and hypertension [58,59]. Importantly, when compared with population normal values, the decrements QoL in GERD patients were independent of whether patients have erosive or nonerosive disease [60].

Some literature reports have highlighted that the presence of mucosal injury has little impact on how reflux symptoms affect individual quality of life. This result is in line with the observation that patients with symptomatic GERD (without any mucosal lesion) experience symptoms that are comparable to those of patients with erosive GERD [61]. Numerous studies have also revealed that the impact on the QoL is often proportional to symptom improvement, and that improvements in QoL in response to treatment are independent of whether esophagitis is present or not [62]. According to our results, we may speculate that the different QoL perception is not only related to the prevalence of GERD-related symptoms. Some reports describe a reduced QoL in subjects with dietary habits based on a Western diet. Moreover, a healthy Mediterranean diet-lifestyle was associated with a lower risk of depression onset [63,64], especially when it was compared with a Western dietary style including processed foods, meat, and dairy, which seems to be associated with an increased risk of depression [65,66]. Accordingly, some randomized control trials described an improvement in depression-related symptom scores when subjects changed from an unhealthy diet (Western) to a healthy diet based on plant foods [63,67,68].

The main strength of this study is the large sample: 1077 questionnaires on GERD-related symptoms and SF-36 were received, in addition to questionnaires about food choices; almost 40% of those who took part in the study declared to follow a vegan diet. Such a large sample, with 402 participants following a diet based exclusively on plant foods, is larger than that of other studies, and provides results with a higher strength of evidence.

Some limitations are also present in this study: data collection relied on self-reported data, thus resulting in possible recall bias and a biased interpretation of the questions. In addition, the design of the study was cross-sectional, which does not allow for the identification of causal relationships. The study was conducted in Italy, hampering the generalizability of the findings to other countries. Moreover, despite the large sample size of participants in the INVITA study (n = 4352), the percentage of those who completed the GERD survey and the SF-36 assessment was relatively low (24.7%). The comparison between those who completed GERDQ and SF-36 (n = 1077) and those who did not complete them (n = 3275) showed that completers were slightly older, more often vegans, and had a lower alcohol consumption. The comparison between the whole INVITA sample (n = 4352) and the Italian general population (≥18 years) showed that there are differences in some characteristics: gender (females 92.2% vs. 51.2%), BMI (>25 kg/m^2^ 17.5% vs. 43%), age (<50 56.3% vs. 85.2%), education (university degree: 52.7% vs. 22.4%), smoking habit (10.1% vs. 24.2%), and ‘at risk’ alcohol consumption (7.0% vs. 17.3%). The vegan dietary pattern, as mentioned above, was over-represented (33.1% vs. 2.4%) [69,70]. All in all, in our study, GERD was defined based on the presence of typical symptoms according to the Montreal Consensus [23] and cannot be considered an objective diagnosis of GERD. Anyway, both versions of the Lyon Consensus [31,71] suggest that typical symptoms are associated with a high likelihood of having objective GERD, corroborating the use of a short course of PPIs in primary care. Finally, the dietary pattern classification in ‘vegan’ vs. ‘non-vegan’ did not permit an evaluation of the quality of the diet.

## 5. Conclusions

In conclusion, this study confirmed that a plant-only (vegan) diet is associated with a lower risk of GERD-related symptoms and could therefore prevent the onset of GERD. The results about quality of life (SF-36 questionnaire, QoL) have shown how the GERD+ participants had a lower score on the SF-36 questionnaire in comparison to GERD− participants. These findings suggest that GERD subjects have a lower perception of their health status, stressing the impact of this disease on the QoL. Considering the low level of evidence of guidelines in suggesting the avoidance of some type of food as a first-line therapy of this disease, the possibility of following a vegan diet, or at least of decreasing the consumption of animal foods, is worthy of consideration as a first-line therapy approach.

## Figures and Tables

**Table 1:** Socio-demographic characteristics of (a) life habits, (b) health-related quality of life (SF-36), and (c) of the overall sample, and of GERD+ and GERD− participants (n = 1077).

a. Socio-Demographic Characteristics	Overall Sample n = 1077	GERD− n = 982 (91.2%)	GERD+ n = 95 (8.8%)	*p*-Value
Gender, n (%)				0.672 Fisher
Male	75 (7.0%)	70 (7.1%)	5 (5.3%)	
Female	1002 (93.0%)	912 (92.9%)	90 (94.7%)	
Age, mean (SD)	37.1 (12.0)	37.0 (11.9)	37.7 (12.9)	0.583 *t* test
BMI, mean (SD)	22.2 (3.8)	22.0 (3.5)	24.1 (5.4)	<0.001 *t* test
Marital status, n (%)				0.508 Fisher
Married	664 (61.7%)	602 (61.3%)	62 (65.3%)	
Not married	413 (38.3%)	380 (38.7%)	33 (34.7%)	
Education, n (%)				0.027 Fisher
Professional qualification/Diploma	362 (33.6%)	321 (32.7%)	41 (43.2%)	
Degree/Post-degree	715 (66.4%)	661 (67.3%)	54 (56.8%)	
Occupation, n (%)				0.097 Fisher
Employed	765 (71.0%)	705 (71.8%)	60 (63.2%)	
Not employed	312 (29.0%)	277 (28.2%)	35 (36.8%)	
**b. Life habits**	**Overall Sample** **n = 1077**	**GERD−** **n = 982 (91.2%)**	**GERD** **+** **n = 95 (8.8%)**	***p*-value**
Dietary pattern, n (%)				0.005 Fisher
Vegan	402 (37.3%)	379 (38.6%)	23 (24.2%)	
Non-vegan	675 (62.7%)	603 (61.4%)	72 (75.8%)	
Monthly alcohol consumption, n (%)	32 missing	30 missing	2 missing	0.864 Chi-square
No consumption	224 (21.4%)	206 (21.6%)	18 (19.4%)	
Low/Moderate ^1^	770 (73.7%)	700 (73.5%)	70 (75.3%)	
At risk ^2^	51 (4.9%)	46 (4.8%)	5 (5.4%)	
Currently smoking, n (%)	5 missing	4 missing	1 missing	0.022 Fisher
No	975 (91.0%)	896 (91.6%)	79 (84.0%)	
Yes	97 (9.0%)	82 (8.4%)	15 (16.0%)	
**c. Health-related quality of life (SF-36), mean (SD)**	**Overall Sample** **n = 1077**	**GERD−** **n = 982 (91.2%)**	**GERD** **+** **n = 95 (8.8%)**	***p*-value** ***t* test**
General health	68.4 (17.2)	69.7 (16.0)	54.3 (22.5)	<0.001
Physical functioning	94.6 (9.9)	95.2 (8.7)	88.3 (17.4)	<0.001
Role limitations due to emotional problems	58.9 (40.9)	60.0 (40.7)	48.2 (41.5)	0.007
Bodily pain	82.7 (20.3)	84.0 (19.4)	68.4 (23.4)	<0.001
Emotional well-being	65.8 (17.1)	66.6 (16.7)	57.9 (18.7)	<0.001
Role limitations due to physical health	84.6 (28.8)	86.0 (27.2)	70.0 (39.4)	<0.001
Energy/fatigue	53.8 (18.4)	54.8 (17.9)	43.9 (20.8)	<0.001
Social functioning	74.3 (22.7)	75.4 (22.0)	63.0 (25.8)	<0.001

^1^ ≤60 alcohol units for males; ≤30 alcohol units for females [26]. ^2^ >60 alcohol units for males; >30 alcohol units for females [26].

**Table 2:** Univariate logistic models for GERD+ participants: unadjusted ORs (n = 1077).

Independent Variable	OR (Unadjusted)	95% CI	*p*-Value
Gender			
Male	Ref.	-	-
Female	1.38	0.54–3.51	0.497
Age	1.01	0.99–1.02	0.345
BMI	1.12	1.07–1.17	<0.001
Marital status			
Married	Ref.	-	-
Not married	0.84	0.54–1.31	0.449
Education			
Professional qualification/Diploma	Ref.	-	-
Degree/Post-degree	0.64	0.42–0.98	0.040
Occupation			
Employed	Ref.	-	-
Not employed	1.48	0.96–2.30	0.078
Dietary pattern			
Non-vegan	Ref.	-	-
Vegan	0.51	0.31–0.83	0.006
Monthly alcohol consumption			
No consumption	Ref.	-	-
Low/Moderate ^1^	1.14	0.67–1.96	0.625
At risk ^2^	1.24	0.44–3.52	0.681
Currently smoking			
No	Ref.	-	-
Yes	2.07	1.14–3.77	0.016
General health	0.96	0.95–0.97	<0.001
Physical functioning	0.98	0.98–0.99	<0.001
Role limitations due to emotional problems	0.99	0.98–0.99	0.008
Bodily pain	0.97	0.96–0.98	<0.001
Emotional well-being	0.97	0.96–0.98	<0.001
Role limitations due to physical health	0.96	0.94–0.97	<0.001
Energy/fatigue	0.97	0.96–0.98	<0.001
Social functioning	0.98	0.97–0.99	<0.001

^1^ ≤60 alcohol units for males; ≤30 alcohol units for females [26]. ^2^ >60 alcohol units for males; >30 alcohol units for females [26].

**Table 3:** Multivariate logistic model for GERD+ participants: adjusted ORs (only independent variables significantly associated at *p* < 0.05 in univariate logistic regression models entered the multivariate logistic regression model).

Independent Variable	OR (Adjusted)	95% CI	*p*-Value
BMI	1.07	1.02–1.13	0.007
Education			
Professional qualification/Diploma	Ref.	-	-
Degree/Post-degree	0.74	0.46–1.19	0.219
Dietary pattern			
Non-vegan	Ref.	-	-
Vegan	0.47	0.28–0.81	0.006
Currently smoking			
No	Ref.	-	-
Yes	1.97	1.03–3.74	0.039
General health	0.97	0.96–0.99	0.001
Physical functioning	1.00	0.99–1.01	0.721
Role limitations due to emotional problems	1.00	0.99–1.01	0.317
Bodily pain	0.98	0.97–0.99	0.005
Emotional well-being	0.99	0.97–1.01	0.544
Role limitations due to physical health	1.00	0.98–1.02	0.921
Energy/fatigue	0.99	0.97–1.01	0.547
Social functioning	1.00	0.98–1.01	0.583
Number of observations	1077		
LR test, *p*-value	Chi2(12) = 94.45, *p* < 0.001		
Hosmer—Lemeshow goodness-of-fit (10 groups)			
Chi2(df), *p*-value	Chi2(8) = 7.55, *p* = 0.479		
Pearson goodness-of-fit			
Number of covariate patterns	1072		
Chi2(df), *p*-value	Chi2(1059) = 1060.64, *p* = 0.480		
Area under ROC curve	0.78		

## Data Availability

The data presented in this study are available on the request from the corresponding author. The data are not publicly available due to privacy law.
